# Spatial and temporal dimensions of landscape fragmentation across the Brazilian Amazon

**DOI:** 10.1007/s10113-017-1120-x

**Published:** 2017-02-27

**Authors:** Isabel M. D. Rosa, Cristina Gabriel, Joāo M. B. Carreiras

**Affiliations:** 1 Life Sciences Department, Imperial College of London, Silwood Park Campus, Buckhurst Road, Ascot, SL5 7PY UK; 2 Centro de Ecologia Aplicada Prof. Baeta Neves, Instituto Superior de Agronomia, Tapada da Ajuda, 1349 - 017 Lisbon, Portugal; 3 National Centre for Earth Observation (NCEO), University of Sheffield, Hicks Building, Hounsfield Road, Sheffield, S3 7RH UK; 4 Biodiversity Conservation Group, German Centre for Integrative Biodiversity Research (iDiv), Deutscher Pl. 5E, 04103 Leipzig, Germany

**Keywords:** Fragmentation, Brazilian Amazon, Deforestation, Forest regeneration, Land use, Conservation, Settlements

## Abstract

The Brazilian Amazon in the past decades has been suffering severe landscape alteration, mainly due to anthropogenic activities, such as road building and land clearing for agriculture. Using a high-resolution time series of land cover maps (classified as mature forest, non-forest, secondary forest) spanning from 1984 through 2011, and four uncorrelated fragmentation metrics (edge density, clumpiness index, area-weighted mean patch size and shape index), we examined the temporal and spatial dynamics of forest fragmentation in three study areas across the Brazilian Amazon (Manaus, Santarém and Machadinho d’Oeste), inside and outside conservation units. Moreover, we compared the impacts on the landscape of: (1) different land uses (e.g. cattle ranching, crop production), (2) occupation processes (spontaneous vs. planned settlements) and (3) implementation of conservation units. By 2010/2011, municipalities located along the Arc of Deforestation had more than 55% of the remaining mature forest strictly confined to conservation units. Further, the planned settlement showed a higher rate of forest loss, a more persistent increase in deforested areas and a higher relative incidence of deforestation inside conservation units. Distinct agricultural activities did not lead to significantly different landscape structures; the accessibility of the municipality showed greater influence in the degree of degradation of the landscapes. Even with a high proportion of the landscapes covered by conservation units, which showed a strong inhibitory effect on forest fragmentation, we show that dynamic agriculturally driven economic activities, in municipalities with extensive road development, led to more regularly shaped, heavily fragmented landscapes, with higher densities of forest edge.

**Electronic supplementary material:**

The online version of this article (doi:10.1007/s10113-017-1120-x) contains supplementary material, which is available to authorized users.

## Introduction

Several studies have shown that land colonisation and land use activities, which can be perpetrated by different agents, lead to distinct spatial patterns in the landscape (e.g. Godar et al. 2014, Wang and Caldas 2014) and patch size distributions over time (Rosa et al. 2012). Understanding how landscapes evolved through time as a result of different land use activities, policies and anthropogenic pressures is essential to identify potential ecological impacts, such as biodiversity and habitat loss (Laurance et al. 2011), and to support efficient policy design (Wang and Caldas 2014). Furthermore, it is critical to know how the spatial pattern of conversion emerged over time, as both patchiness and total amount of remaining habitat can have distinct impacts on biodiversity (Fahrig 2013), and carbon storage can vary by twofold to fourfold depending on the emerging pattern (Chaplin-Kramer et al. 2015).

One of the consequences of forest disturbance is the fragmentation of the landscape, leading to a mixture of land cover patches of different classes, sizes and shapes (Numata et al. 2011). Landscape fragmentation induces major changes in the equilibrium of the ecosystems (Haddad et al. 2015), such as fundamental alterations to the aboveground biomass dynamics (Nascimento and Laurance 2004), marked differences in species richness and abundance (Ochoa-Quintero et al. 2015) and extinction of native biota (Pardini et al. 2010).

The Brazilian Amazon has been subjected to extensive deforestation in the past four decades (Davidson et al. 2012). Annual deforestation mapping generated from satellite data has been made publicly available by the Brazilian National Institute for Space Research (Instituto Nacional de Pesquisas Espaciais, INPE) since 1988 within the scope of the PRODES project (INPE 2013). Reported deforestation rates have been highly variable over time, reaching its highest value in the mid-1990s (~30,000 km^2^ yr^−1^), but decreased progressively since the mid-2000s up to a record low of ~4500 km^2^ yr^−1^ in 2012. These fluctuations in the deforestation rate of the Brazilian Amazon can be attributed to the combination of many socio-economic factors, which varied over time (Ewers et al. 2008, Brondizio and Moran 2012). In particular, between the 1970s and 1980s, deforestation was mainly a result of government-sponsored initiatives to colonise the region, which featured extensive road building, granting land titles to settlers and tax incentives (Fearnside 2005). Beginning in the 1990s, and up until today, national and international commodities demand, such as soybeans and beef, started playing a stronger role in the temporal variations of annual deforestation rates (Nepstad et al. 2006; Laurance 2007). The unsustainable rates of deforestation observed in the late 1990s and early 2000s were successfully reduced by a combination of stronger law enforcement (e.g. command and control operations), expansion of the conservation units’ network and the implementation of the best forest monitoring system in the world (Nepstad et al. 2014). These were also helped by an international economic crisis as well as by fair-trade initiatives to prevent the commercialisation of products that led to illegal deforestation in the Brazilian Amazon, such as the soy moratorium (Gibbs et al. 2015). Most of the deforested area is currently under agricultural use, especially for cattle ranching; however, land abandonment has occurred in many areas, thus allowing the expansion of secondary succession forest of different ages (Davidson et al. 2012).

Landscape metrics have often been used to evaluate forest fragmentation and land cover patterns over time in many environments and regions (e.g. Lung and Schaab 2006; Weng 2007; Peng et al. 2010). Particularly in the Brazilian Amazon, Wang and Caldas (2014) used multi-temporal Landsat data and three landscape metrics to show how different types of settlements in the state of Pará—spontaneous colonisation versus social movement organisation-led settlements—impacted forest fragmentation over time. Similarly, Batistella et al. (2003) used these metrics to investigate land change in two adjacent settlements in Rondônia and understand how their different designs impacted the landscape. At a coarser scale, Colson et al. (2011) quantified and investigated the spatial patterns of two land uses based on eight landscape metrics, in the states of Pará, Mato Grosso, Rondônia and Amazonas. Finally, using data that covered two decades of deforestation in Rondônia, Frohn and Hao (2006) evaluated the performance of sixteen landscape metrics at different spatial scales.

In the Brazilian Amazon, fragmentation has been mainly a result of anthropogenic activities, such as road building, logging, or clearing land for agriculture activities and associated occurrence of wildfires, and to a much lesser extent the occurrence of natural events such as blowdowns (Ahmed et al. 2013; Aragão and Shimabukuro 2010, Brando et al. 2014; Espírito-Santo et al. 2005). Taking advantage of having quasi-annual land cover maps spanning nearly 30 years, and classified into mature forest, non-forest and secondary forest classes, on different states of the Amazon (Carreiras et al. 2014), the main goal of this study was to examine the size, shape and aggregation of land cover patches undergoing distinct rates and fragmentation patterns over time. In particular, we investigated the temporal and spatial dimensions of landscape fragmentation across three study areas: Manaus (Amazonas state), Santarém (Pará) and Machadinho d’Oeste (Rondônia), comparing the dynamics inside and outside conservation units. Despite a strong human presence in all regions, these landscapes were colonised differently, the land use activities practised in them were distinct over time, and conservation activities were also different. As such, we make inferences and associate the observed fragmentation matrices with land use activities (e.g. cattle ranching, crop production), occurrence of large-scale wildfires, occupation processes (e.g. spontaneous, planned settlement), as well as the implementation and effectiveness of conservation units.

## Methods

### Study areas

The Manaus site has an area of ~5000 km^2^ (Figure S1) and encompasses the majority of a federal conservation unit: Biological Dynamics of Forest Fragments Project (BDFFP) (Laurance et al. 2011); other county and state conservation units were also included in this study area. The construction of a highway connecting Manaus with Boa Vista (BR-174) in the early 1970s caused the inception of deforestation in the region and subsequent agricultural expansion. In 1979, several forest fragments inside what is now the BDFFP were preserved, prior to deforestation of the surrounding forest, and used to study the impacts of deforestation on ecosystem structure and function (e.g. Uriarte et al. 2011), to inform future conservation programmes in the Amazon (Laurance et al. 2011).

The Santarém site, with ~1100 km^2^ (Figure S1), is partially within a federal conservation unit—Tapajós National Forest—between the Tapajós River and the BR-163 highway connecting Santarém with Cuiabá (Mato Grosso). This unit was created in 1974 and has been used successfully to implement novel forest management practices, such as the benefits of reduced impact logging on social welfare and biodiversity (e.g. Bacha and Rodriguez 2007; van Gardingen et al. 2006).

The Machadinho d’Oeste site, with an area of ~1800 km^2^, is mainly located within the Machadinho d’Oeste municipality (Figure S1). Its origins are a settlement project, deployed by the Brazilian federal government in 1982 to colonise the Amazon (Miranda 2013). The original vegetation is dominated by open rainforests (Miranda 2013), and according to Batistella and Moran (2005), most of its inhabitants live from subsistence agriculture. This site includes several state-level conservation units, mainly extractive reserves, which were implemented in the mid-1990s.

In terms of land cover (Table S1), mature forest was the dominant class in all three regions at the beginning of the time series: in 1984/1985 mature forest covered 90.7% of the Machadinho d’Oeste landscape, 78.8% of Santarém and 83.3% of Manaus landscape (Carreiras et al. 2014). Over time, mature forest declined significantly, and by the end of the time series, in 2010/2011, Santarém and Machadinho d’Oeste already had more than 50% of its area covered by either non-forest or secondary forest (Carreiras et al. 2014).

### Remote sensing data and image classification

Time series of 3-class land cover maps (mature forest, non-forest and secondary forest) was obtained from automatic classification of Landsat sensor data over the three selected sites. Carreiras et al. (2014) and Prates-Clark et al. (2009) provide detailed information about the remote sensing data processing and methods used to generate the 3-class land cover maps, as well as information on the post-processing and corresponding accuracy assessment. In this section, only a summary is presented, for more details please see the above-mentioned publications.

In Manaus, Prates-Clark et al. (2009) and Carreiras et al. (2014) used Landsat Multi-spectral Scanner (MSS), Thematic Mapper (TM) and Enhanced Thematic Mapper Plus (ETM+) data acquired in the periods 1973–2003 and 2006–2011, respectively, to analyse the land cover dynamics in the region. In Santarém, Landsat TM data were acquired between 1984 and 2003 (Prates-Clark et al. 2009) and in the period 2005–2010 (Carreiras et al. 2014), and then classified to generate the time series of 3-class land cover maps. In Machadinho d’Oeste, Landsat TM data from the period 1984–2011 were used to create the time series of 3-class land cover maps (Carreiras et al. 2014). To maintain consistency among the three time series, only data from 1984 onwards were used in this study (Table S2). At all sites, most scenes were unaffected by substantive cloud cover and overall gaps in the time series ranged from one (70%) to four years (3%). Several parametric (minimum distance, maximum likelihood) and nonparametric (random forests) classification algorithms were used with the objective of generating the best possible discrimination among the three land cover classes at all three sites.

### Fragmentation metrics

We used FRAGSTATS 4 (McGarigal et al. 2012) to investigate how fragmentation emerged over time in each of our three study areas, separately for areas inside versus outside conservation units, assessing the impact of the occupation process and land use dynamics. Since many landscape metrics are redundant and statistically correlated, we chose a parsimonious set of uncorrelated metrics to use (described below) that capture landscape configuration (e.g. area, shape) and aggregation.

The scale of our analyses was focused at the class level, because we were interested in investigating changes over time on the three existing land cover classes (mature forest, non-forest and secondary forest classes), rather than changes at the patch level. Determining metrics at the class-level implies the integration of all patches of a given class. The statistic used to combine all patches is specified below where we detailed the chosen metrics. We used the eight-cell neighbours to assign a given pixel to a particular patch.

Within each site and for each land cover class, we fitted linear regressions to test for positive/negative trends over time on the metrics’ values, taking into account temporal autocorrelation. These analyses were performed in R (R Core Team 2013) using the *auto.arima* function to determine the temporal autocorrelation structure within the data and then the *gls* function to determine the temporal structure. Time was the only independent variable for each regression that was fitted per fragmentation metric (dependent variable), per landscape (inside or outside conservation units) and per land cover in each landscape.

#### Edge density (ED)

Edge effects are some of the most important drivers of ecological change (Laurance et al. 2011). To allow a straightforward comparison across sites with different areas, we chose edge density (ED) rather than total edge. ED is determined by summing the lengths (m) of all edge segments of the patches within each land cover class. This is then divided by the total landscape area.

#### Clumpiness index (CLUMPY)

This is a normalised index that indicates whether a certain land cover class is aggregated or dispersed across the landscape. CLUMPY values can fluctuate between −1 (maximally disaggregated) and 1 (maximally clumped). If zero, the patch distribution is no different than random. Values under zero suggest greater dispersion (or disaggregation), whereas values greater than zero suggest a more clumped landscape. This metric is important to understand spatial connectivity across forest patches, for example, which has strong implications on species conservation (Donald and Evans 2006).

Finally, to investigate patch size and shape distributions in our study sites we determined the area-weighted version of these metrics, respectively, the area-weighted mean patch size (AREA_AM) and area-weighted mean patch shape index (SHAPE_AM). In both cases, the area-weighted mean of each index is given by the sum across all patches constituting each land cover class, weighted by the proportional abundance of the patch (McGarigal et al. 2012).

#### Area-weighted mean patch size (AREA_AM)

This index gives the area-weighted mean patch size of all the patches within each land cover class, where the proportional area of each patch is based on total land cover class area, i.e. the total area of mature forest, secondary forest and non-forest. We have considered this metric so we could measure the degree of fragmentation of each land cover class, i.e. whether there were many small patches or whether the landscape was dominated by large patches, and how that varied, over time, across land covers and landscapes.

#### Area-weighted mean patch shape index (SHAPE_AM)

This metric is given by the sum of the patch shape index across all patches of each land cover class, multiplied by the proportional abundance of the patch. If the value is 1, the patch is essentially a square, and values increasingly higher than one result in patches increasingly irregular. We included this metric to evaluate how different anthropogenic activities and/or natural events could lead to more regularly or irregularly shaped landscapes, and again how that varied, over time, across land covers, inside and outside conservation units.

### Relative incidence of deforestation inside and outside conservation units

Conservation units (CUs) play a very important role in preventing further deforestation in the Brazilian Amazon (Soares-Filho et al. 2010), but with the observed rise in anthropogenic pressures over the last decades, they have become more isolated, and their role increasingly threatened (DeFries et al. 2005). We tested whether these observations were also valid in our study areas, by determining the relative incidence of deforestation (RID) across time, both inside and outside existing CUs, given by:

1 $${\text{RID}} = \frac{{\left( {\frac{{D_{i} }}{{D_{\text{total}} }}} \right)}}{{\left( {\frac{{A_{i} }}{{A_{\text{total}} }}} \right)}}$$

where* D*_i_ is the deforested area of class *i* (CU or non-CU), *D*_total_ is the total deforested area, *A*_*i*_ is the area of class *i*, and *A*_total_ is the total area. Values under 1 mean that deforestation in a given class is less than its share in the landscape, whereas values above 1 mean that deforestation in that class is higher than its share in the landscape. RID was used to normalise the incidence of deforestation inside and outside CUs by their respective areas, which is relevant since CU and non-CU areas are different at each site and also among sites. Then, we tested for its significance over time similarly to what we did for the fragmentation metrics. Fluctuations in RID over time inform us on whether these units in the three regions under analysis have, in fact, been suffering higher anthropogenic pressure in recent years.

## Results

**Fig. 1:**
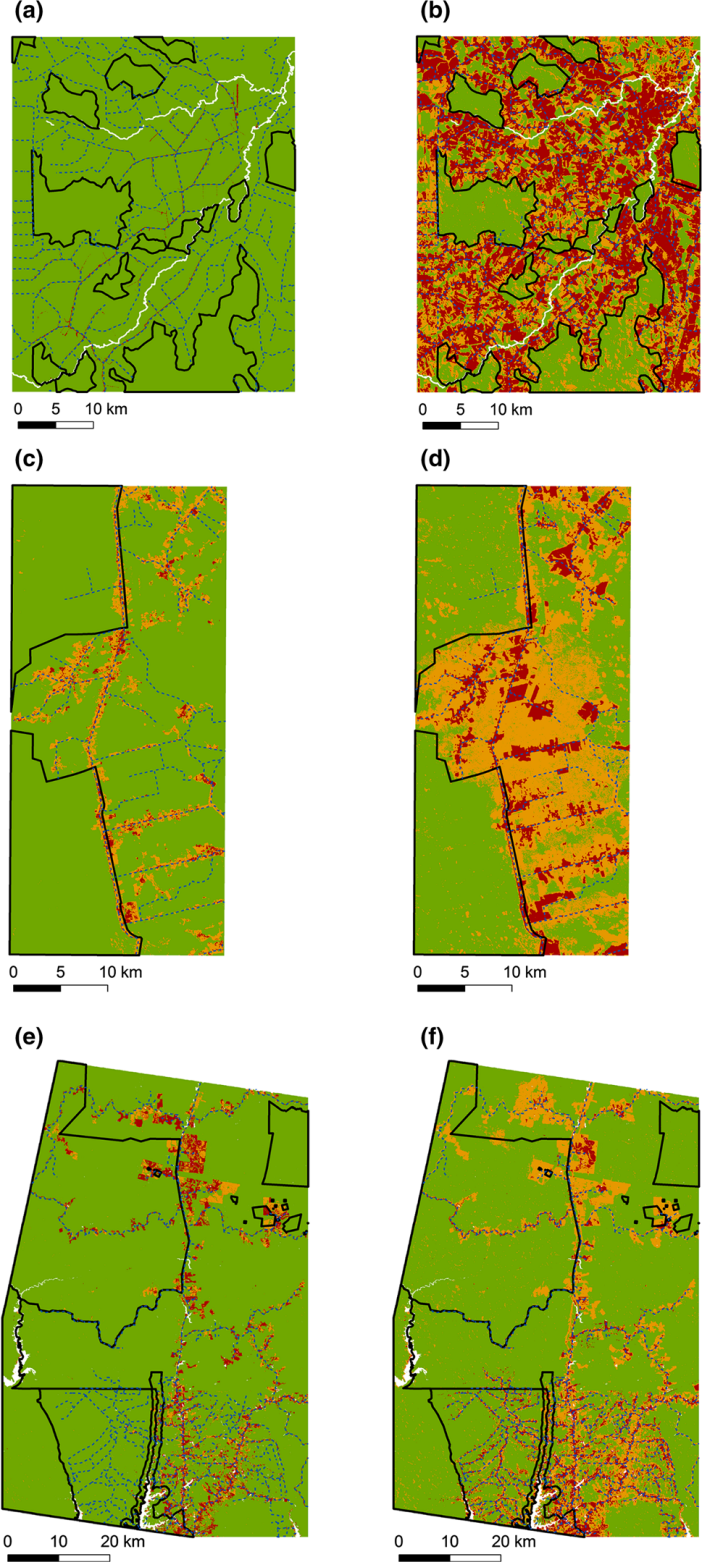
Land cover (mature forest—*green*, non-forest—*red* and secondary forest—*orange*) at the beginning and by the end of our time period of analysis in Machadinho d’Oeste: **a** 1984 and **b** 2011, Santarém: **c** 1984 and **d** 2010, and Manaus: **e** 1985 and **f** 2011. *Black solid lines* show the boundaries of the conservation units; *blue dashed lines* show the road network in 2010

Santarém and Manaus started the time series with a (proportionally) larger area of secondary and non-forest compared to Machadinho d’Oeste (21.2% and 16.7% vs. 9.3%), which was almost intact before the 1980s (90.7% mature forest) (Table S1). Nonetheless, in all three landscapes we observed significant changes in land cover between the first and last time step of the time series analysed (Fig. 1). By the end of the time series, mature forest in Machadinho d’Oeste occupied only 31.6% of the landscape, 55.7% of which within conservation units (more than doubling the initial proportion of 23.4%). Santarém ended the time series with about 46.2% of mature forest, 62.2% of which inside CUs, whereas Manaus, by contrast, reached 2011 with 71.9% of mature forest evenly distributed inside and outside CUs.

### Fragmentation metrics

#### Edge density (ED)

**Fig. 2:**
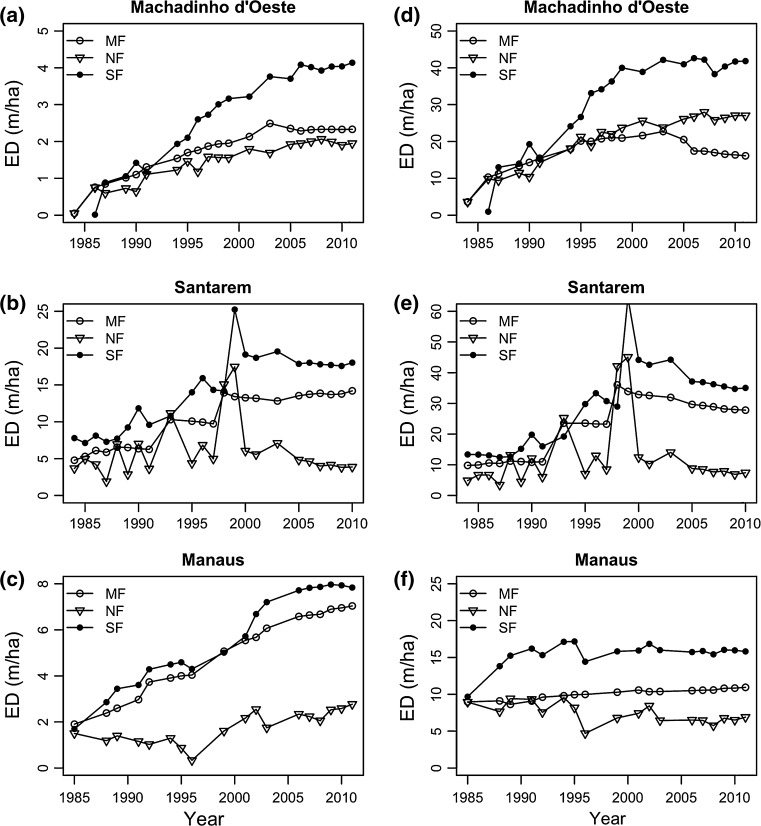
Edge density (ED) calculated for each land cover class [mature forest (MF), non-forest (NF), and secondary forest (SF)] in Machadinho d’Oeste, Santarém and Manaus between 1984 and 2011, **a**–**c** inside and **d**–**f** outside conservation units

Edge density in mature forest increased significantly over time in all landscapes, both inside and outside CUs (Fig. 2, Table S3). ED was higher outside CUs than inside in both Machadinho d’Oeste and Santarém, and the steepest increase was observed in Santarém. Here, ED varied from a minimum of 4.8 m/ha in 1984 (inside CUs) to a maximum of 36.0 m/ha in 1998 (outside CUs), whereas in Machadinho d’Oeste it varied between 0.05 m/ha (inside CUs) in 1984 and 22.7 m/ha in 2003 (outside CUs), and in Manaus from 1.9 m/ha (inside CUs) in 1985 to 10.9 m/ha (outside CUs) in 2011 (Fig. 2). The pattern is inverted in Manaus, where ED increased more inside than outside conservation units.

ED in the non-forest class has only significantly increased over time in Machadinho d’Oeste (Table S3), both inside and outside CUs, and in Manaus, only inside CUs (since it decreased significantly outside). In Machadinho d’Oeste, ED varied from a minimum of 0.05 m/ha inside CUs in 1984 to a maximum of 27.0 m/ha in 2007 (Fig. 2), outside CUs. Figure 2 shows two peaks in ED values for Santarém in 1993 and 1998 that match the occurrence of two large wildfires.

ED in secondary forest class has also increased significantly over time outside CUs in Machadinho d’Oeste and Santarém (Table S3). In Machadinho d’Oeste, this metric varied from a minimum of 0.01 m/ha (inside CUs) in 1986 to a maximum of 41.8 m/ha in 2011 (outside CUs), whereas in Santarém it varied between 7.8 m/ha in 1987 and 64.4 m/ha (the peak observed for 1999, outside CUs, in Fig. 2).

In all three regions, and for all three land cover classes, this metric’s value was always lower inside CUs than outside throughout the time series, and it increased over time (except for NF in Manaus). Machadinho d’Oeste showed the highest magnitudes of changes in ED for all three classes, both inside and outside CUs.

#### Clumpiness index (CLUMPY)

**Fig. 3:**
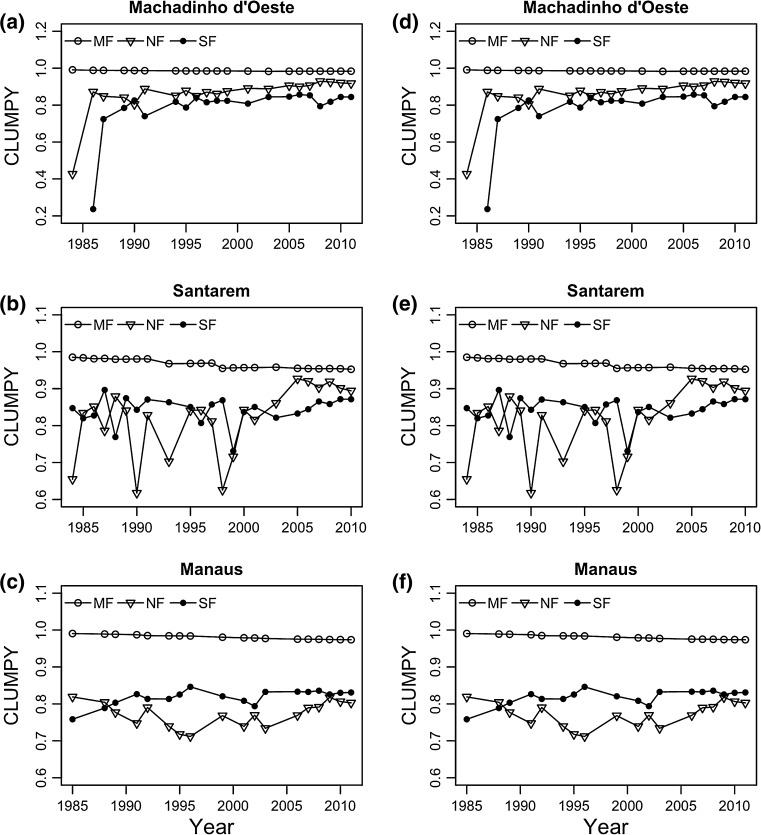
Clumpiness index (CLUMPY) determined for each land cover class [mature forest (MF), non-forest (NF), and secondary forest (SF)] in Machadinho d’Oeste, Santarém and Manaus between 1984 and 2011, **a**–**c** inside and **d**–**f** outside conservation units

Clumpiness Index (CLUMPY) values were always greater than 0.2 throughout the time period, in all three regions, and for all three land cover classes (Fig. 3), inside and outside CUs. Both inside and outside CUs, CLUMPY in mature forest decreased significantly over time in Machadinho d’Oeste, from 0.97 in 1984 to 0.89 in 2011 (outside CUs) and from 0.99 in 1984 to 0.98 in 2011 (inside CUs); in Manaus, from 0.97 in 1985 to 0.96 in 2011, and from 0.99 in 1985 to 0.97 in 2011 (inside CUs); and in Santarém, from 0.97 in 1984 to 0.85 in 2010, and from 0.99 in 1985 to 0.95 in 2010 (inside CUs).

In Machadinho d’Oeste and Santarém, both inside and outside CUs, our results showed a significant increase in CLUMPY in non-forest (Table S3), contrarily to Manaus where the relationship was non-significant. Strong oscillations for this parameter were observed in Santarém (Fig. 3). The CLUMPY values in Machadinho d’Oeste varied between 0.59 in 1984 and 0.91 in 2011 (outside CUs) and from 0.43 to 0.92 inside CUs, whereas in Santarém these varied between 0.69 in 1984 and 0.91 in 2010, outside CUs, and from 0.65 to 0.89 inside CUs, for the same years (Fig. 3).

In Machadinho d’Oeste and Manaus, both inside and outside CUs, we observed an increase in the values of CLUMPY in secondary forest (Table S3), which is consistent with the values obtained for non-forest, which are necessarily linked because the occurrence of secondary forest needs to be preceded by non-forest. This metric varied from 0.38 in 1986 to 0.82 in 2011, outside CUs, and from 0.24 to 0.84 inside CUs in Machadinho d’Oeste; in Manaus from 0.72 in 1985 to 0.87 in 2011 outside CUs and from 0.76 to 0.83 inside CUs.

For the majority of the time steps (69%) in the time series analysed, CLUMPY values were higher inside CUs than outside, for all three land cover classes and in all three regions. This was particularly true for secondary forest (78%) and mature forest (75%).

#### Area-weighted mean patch size (AREA_AM)

**Fig. 4:**
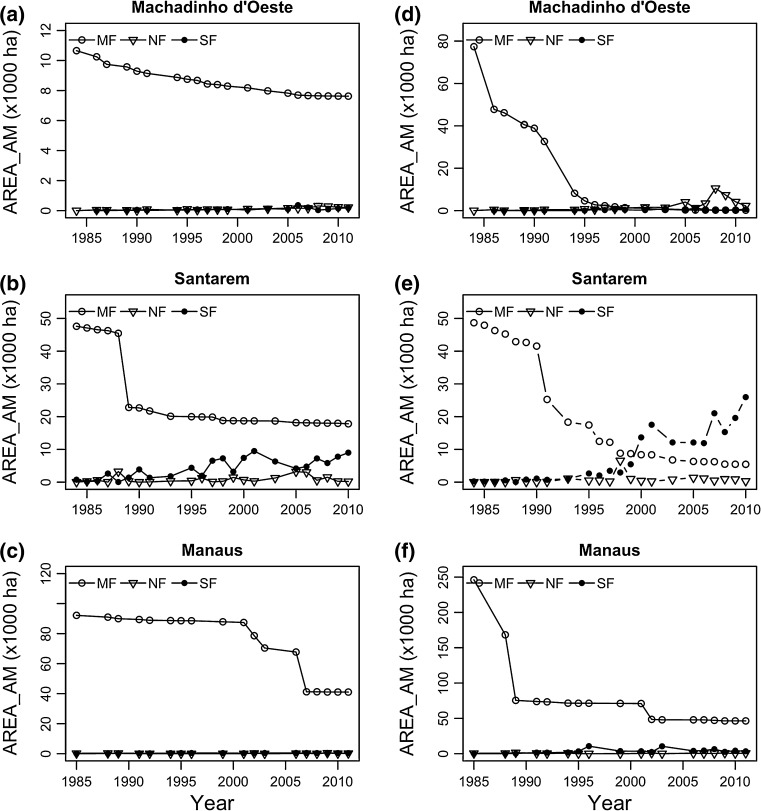
Area-weighted mean patch size area (AREA_AM) for each land cover class [mature forest (MF), non-forest (NF), and secondary forest (SF)] in Machadinho d’Oeste, Santarém and Manaus between 1984 and 2011, **a**–**c** inside and **d**–**f** outside conservation units

At the beginning of the time period covered in this study, the largest patches belonged to mature forest in the three landscapes (Fig. 4). However, by the end of the time period that was still true only for Manaus. In all three regions, a significant and sharp reduction in the AREA_AM values was observed for this land cover class (Table S3), both inside and outside CUs. The steepest reductions were observed outside CUs: in Manaus, with an AREA_AM of 246,122 ha in 1985 and 46,415 ha by 2011; followed by Machadinho d’Oeste, with the values of AREA_AM declining from 77,375 ha in 1984 to 211.7 ha in 2011; and Santarém, where AREA_AM varied from 48,672 ha in 1984 to 5446 ha in 2010 (Fig. 4).

Conversely, we found a significant positive trend in the AREA_AM of secondary forest in all three regions, both inside and outside CUs, although higher outside. This increase was particularly sharper in Santarém, where the AREA_AM index varied from 231.8 ha in 1984 to 25,956 ha in 2010; followed by Manaus and Machadinho d’Oeste, where AREA_AM varied between approximately 168.4 and 1.4 ha in 1985/1986 to 3347 and 633.1 ha in 2011, respectively. Regarding non-forest, only in Machadinho d’Oeste, both outside and inside CUs, we found a significant increase in the AREA_AM values over time (Table S3).

#### Area-weighted mean patch shape index (SHAPE_AM)

**Fig. 5:**
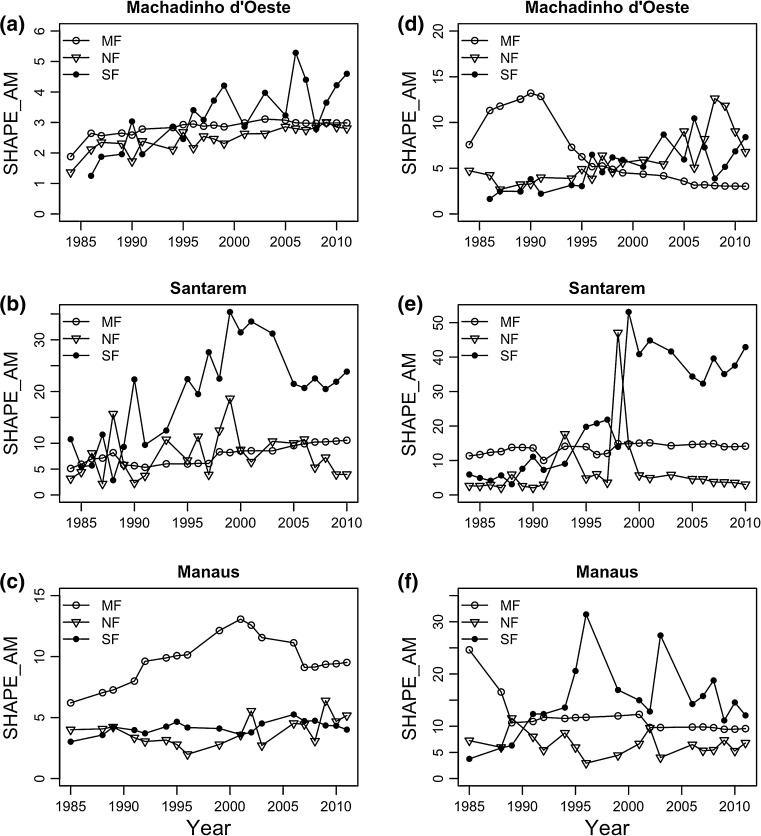
Area-weighted mean patch shape index (SHAPE_AM) for each land cover class [mature forest (MF), non-forest (NF), and secondary forest (SF)] in Machadinho d’Oeste, Santarém and Manaus between 1984 and 2011, **a**–**c** inside and **d**–**f** outside conservation units

The results obtained for this metric showed a less clear trend for all three regions (Fig. 5). For mature forest, outside CUs, there was a significant decrease in the values of SHAPE_AM both in Machadinho d’Oeste and Manaus. In Manaus, this metric changed from a maximum of 24.6 in 1985 and a minimum of 9.6 in 2011, whereas in Machadinho d’Oeste it oscillated between 7.6 in 1984, reaching a maximum of 13.2 in 1990, and a minimum value of 3.0 by the end of the time period. The opposite trend was observed in Santarém (Table S3), where this metric varied between a minimum of 10.0 in 1991 and a maximum of 15.1 in 2001, ending the time period at a value of 14.2.

With regard to non-forest and secondary forest, in Machadinho d’Oeste we found significant trends (Table S3), with both becoming increasingly irregular over time inside and outside CUs. On the other hand, in Santarém, only secondary forest was significantly more irregular over time, both inside and outside CUs. In both regions, irregularity was higher outside than inside CUs. No significant trend was observed neither in Manaus (non-forest and secondary forest) nor in non-forest in Santarém, due to the strong fluctuations in the value of this metric (Fig. 5).

### Relative incidence of deforestation inside/outside conservation units

By the end of the time period analysed, in Manaus, the area of mature forest comprised 78.0% of the conservation units (CUs) and 67.2% of the outside, whereas in Santarém and Machadinho d’Oeste it encompasses 78.0% and 75.7% of the CUs, respectively, and only 27.6% and 18.2% of the outside (Table S1, Fig. 1). In Santarém, we found an overall significant increase in relative incidence of deforestation (RID) outside CUs (Table S4 and Figure S2), whereas inside it decreased over time. In Manaus, we found that RID increased in the State CU, but decreased in the County and Federal CUs as well as in the areas under no protection (Table S4). Areas that do not have any conservation status in Santarém and Machadinho d’Oeste had much higher RID values than those who have, with the year of implementation not having any impact on this metric. In Machadinho d’Oeste, between the first and last date of the time series, we found that RID inside the State CUs increased by tenfold, whereas in non-CUs it decreased by 5%. Distinct conservation status (federal, very limited activities vs. state, some extraction activities allowed) might help explaining the differences found in the slopes of the fitted regressions (Table S4).

In Manaus, the county CU had the highest RID for the majority of the time period, with a slight increase of 0.7% RID between the beginning and end of the time series (Figure S2). In the federal units, there was a ~50% reduction in RID; however, the high RID observed in the federal CU of Manaus can be explained by the fact that this unit contains the BDFFP research sites (Laurance et al. 2011). Finally, we also determined a 70% increase in RID inside State CUs of Manaus, and a 12% reduction in the RID outside CUs.

## Discussion

### Emerging fragmentation patterns

Although evidence of landscape fragmentation was observed in all landscapes, we found strong differences across regions, as well as over time on the three land cover types within regions. Our findings show that Manaus, which is located in the north-western part of the Amazon, far from the dynamic Arc of Deforestation, was the least fragmented landscape by the end of the time period (Fig. 1). Nonetheless, an increasing edge density (ED) and a strong decrease in the patch area (AREA_AM) indices in the mature forest class (Figs. 2 and 4) indicate that the old-growth forest is indeed being fragmented.

In Santarém and Machadinho d’Oeste, both located in areas where agro-businesses are well established (Barona et al. 2010), and road networks are extensive (Ahmed et al. 2013), mature forest now represents less than 50% the landscape and is almost strictly confined to conservation units (Fig. 1). Further, ED has risen significantly faster in these landscapes compared to Manaus, which is a result of higher deforestation rates. In Machadinho d’Oeste, the value of this metric rose steadily over time, whereas in Santarém we observed several peaks (Fig. 2), most likely to be related to the occurrence of wildfires, associated with agriculture activities, which caused a more scattered pattern of fragmentation (replicated for the CLUMPY and SHAPE_AM metrics). Fire is often used as a tool in agriculture; however, farmers sometimes lose control of it, leading to uncontrolled wildfires (Brando et al. 2014). The occurrence of an El Niño Southern Oscillation event in 1997–1998 promoted extremely dry weather conditions, which helped the propagation of the late 1990s large wildfires. These wildfires tend to leave a more irregularly shaped scar on the landscape than do the anthropogenic activities that lead to deforestation, such as converting forest to pastures and/or croplands.

With regard to patch size distributions (AREA_AM), by the end of the time period, on the contrary with what was observed for Manaus, the secondary forest in Santarém, and the non-forest class in Machadinho d’Oeste, had the largest patches. Such result in Machadinho d’Oeste can be due to either (1) a change in the agents of deforestation or their motivations in the region that now deforest larger areas; or (2) further support to previous findings that deforestation tends to occur next to already deforested areas, leading to larger patches of non-forest over time through coalescence of spatially contiguous patches of deforested land (Rosa et al. 2013, 2015). Regarding Santarém, wildfires were major contributors to the large amount of secondary forest that was generated following these events in the 1990s. However, it is possible that the consequence of these events was not a full conversion from mature to secondary forest, but rather to a mosaic of mature and secondary forest species. From a pure remote sensing point of view, it was difficult to ascertain the degree of damage caused by these wildfires, and therefore, all area was considered to have first transitioned to non-forest (burnt scar) and subsequently to secondary forest (Carreiras et al. 2014).

While the overall pattern of the landscape metrics was similar inside and outside conservation units, showing a forest getting increasingly scattered and larger non-forest patches emerging over time, we still found important differences. In all three regions, fragmentation was more severe outside conservation units with higher increases in ED and steeper reductions in the area of mature forest. Although this suggests that CUs have had a role in preventing further fragmentation, in agreement with previous findings (Barber et al. 2014), our RID analysis in Machadinho d’Oeste, the landscape that has undergone the highest land cover change, has shown that existing CUs are suffering increasing pressure from deforestation activities due to significant reduction in unprotected forested areas.

### Land use dynamics and occupation processes

In the time series analysed, both Santarém and Manaus start off with a higher proportion of secondary forest and non-forest compared to Machadinho d’Oeste, which reflects the fact that these two regions were spontaneously colonised in the 1970s, whereas Machadinho d’Oeste was mainly intact before the 1980s (Table S1). The fact that Manaus was the least fragmented landscape can be explained in part by: (1) its isolation—road density in this municipality is much lower when compared to the municipalities in the Arc of Deforestation (Ahmed et al. 2013); and (2) by a lack of favourable conditions for agriculture—wetter weather and poorer soils (Fearnside 2005). However, the increasing trend in mature forest fragmentation in this region is most likely a result of a rise in agriculture activities. According to the Brazilian Institute of Geography and Statistics (IBGE, http://www.sidra.ibge.gov.br/), especially permanent cultures of coconuts and oranges are increasing in the region (Figure S3). In Manaus, cattle ranching still constitutes a fairly low share of just 0.16% of the municipality’s gross domestic product (Giatti et al. 2014).

Santarém and Machadinho d’Oeste sites are very different from each other, and from the Manaus site, in terms of history of occupation and land use activities. Machadinho d’Oeste was a planned settlement part of a colonisation program implemented by the government in the 1980s (Batistella and Moran 2005). Past studies have shown that creating a new settlement has an immense impact on forest fragmentation (Wang and Caldas 2014), and Machadinho d’Oeste was no different. The people who live there nowadays depend heavily on agricultural activities for income, especially cattle ranching and coffee plantations (Gomes et al. 2009; Miranda et al. 2008). As a result, the number of cattle heads in Machadinho d’Oeste, according to IBGE, rose rapidly from nearly zero in the 1980s to more than 250,000 by 2010, representing a density of 0.31 heads/ha (Figure S3). Further, by 2014 the area dedicated to pastures occupied 40% of the landscape (Ferreira et al. 2014). Such increase came at the cost of forest to establish pastures, a pattern supported by our analyses, which show higher ED, smaller and more irregular mature forest fragments. Additionally, the area harvested of permanent crops has also increased over time (Figure S3), but seems to have been decreasing slightly over the last few years. This could be due to an increased investment on cattle ranching or the lack of forested lands to move into, since most of the remaining mature forest is located within CUs (Fig. 1), which are illegal to deforest.

The history of land use activities in Santarém, on the other hand, is closely related to the BR-163 road, which was built by the government in the 1970s, as part of the Amazon colonisation program. This allowed farmers to spontaneously establish themselves along this road. Initially, farmers in this region were mainly small-scale agrarian reform colonists. However, and despite being illegal, many of these farmers were pressured to sell their land to large-scale agribusinesses (Barros 2010). Land availability, the perspective of paving of the BR-163 road, thus having an easy way to transport their goods, and the existence of a port in the city of Santarém, turned this region into a very attractive location for agriculture activities, and associated unofficial road expansion (Viana and Fonseca 2009; Fearnside 2007). The area harvested of annual crops has risen rapidly since the early 2000s (Figure S3). This pattern is attributed to the fast expansion of soybean production in this municipality, supported by the improvements made on the Santarém port by Cargill (Barros 2010). Such dynamics has significantly impacted the landscape, as our results show, with mature forest becoming fragmented into smaller and more irregular patches, thus resulting in larger edge densities.

In comparison with Santarém, deforestation was more persistent over time in Machadinho d’Oeste, because farmers took advantage of having more fertile soils and higher water availability (Brondizio and Moran 2012). Thus, their focus was on permanent crops (coffee, cocoa and sugar cane) and low cattle ranching investment to maintain their farm profitability (Brondizio and Moran 2012). The last remaining large patches of mature forest are concentrated in the Tapajós National Forest (Fig. 1), but even in this National Forest it was observed an increase in roads, which suggests that colonists are moving inside this CU (Viana and Fonseca 2009), also supported by our findings of an increasing RID inside the Tapajós reserve.

We were expecting to find stronger differences in the landscape metrics for Santarém and Machadinho d’Oeste. In terms of rate of change, Machadinho d’Oeste was the region where the forest was fragmented at a faster pace, which is in line with the findings of Wang and Caldas (2014), who argued that spontaneous settlements, such as the Santarém site, change at a slower rate when compared to planned settlements (the Machadinho d’Oeste site). In terms of spatial configuration of the landscapes, we expected pastures and permanent agriculture to lead to larger, more aggregated, and more regularly shaped landscapes due to the lower need of finding new land for new plantations, as opposed to temporary agriculture which is much more dynamic and would occur on smaller, dispersed, and more irregularly shaped patches. However, overall, the patterns observed in the landscape metrics for Santarém and Machadinho d’Oeste were similar. The greatest differences were attributed to the fire scars left behind by the occurrence of wildfires, associated with agriculture activities, rather than any particular land use policy or change in agricultural production. This is a result supported at a coarser scale by Colson et al. (2011). The authors found that different land use policies led to larger discrepancies in landscape patterns at an early stage; however, agricultural activities and cattle ranching were later responsible for larger similarities across landscapes.

### The importance of understanding fragmentation

In this study, we used a long time series of quasi-annual data of mature forest, non-forest and secondary forest in three municipalities of the Amazon that have been undergoing distinct land cover change and land use activities. Our time series started in the early 1980s and ended in the early 2010s, thus covering all major fluctuations observed in the land cover change dynamics of the Brazilian Amazon (INPE 2013). Understanding fragmentation patterns in the Brazilian Amazon, which is so incredibly diverse, not only in ecological terms, but also in human-related activities, is vital for designing policies that can effectively manage deforestation in the region (Wang and Caldas 2014) and prevent further ecological impacts (Laurance et al. 2011). Policies, such as expansion of CUs network, and market-based initiatives have been successful in reducing deforestation rates in the Brazilian Amazon (Nepstad et al. 2014; Godar et al. 2014). However, it seems that these were only efficient in reducing large-scale deforestation (Rosa et al. 2012), more susceptible to fines and embargos by enforcement agencies (Godar et al. 2014). Further, now that the remaining mature forest, at least in the municipalities along the Arc of Deforestation, is becoming strictly contained within CUs, these will likely suffer stronger pressures from anthropogenic activities, as our results in Machadinho d’Oeste suggest. The differences found between our study sites show that conservation policies designed to prevent further deforestation in the Brazilian Amazon need to account for temporal dynamics and location-specific processes and agents of land cover change, to ensure their success. Having detailed spatially explicit information to assess land use and land management impacts is the first critical step to understand historical changes and to be able to improve conservation policies.

## Electronic supplementary material

Below is the link to the electronic supplementary material.

Supplementary material 1 (DOCX 825 kb)
