# Supplementary material for: Spatial and temporal dimensions of landscape fragmentation across the Brazilian Amazon
Source: Reg Environ Change. 2017 Feb 27;17(6):1687–99. doi: 10.1007/s10113-017-1120-x (PMC5514199; doi:10.1007/s10113-017-1120-x)
Supplement: Supplementary file 1 — Supplementary material 1 (DOCX 825 kb) [file 10113_2017_1120_MOESM1_ESM.docx]

**Supplementary Materials**

**Spatial and temporal dimensions of landscape fragmentation across the Brazilian Amazon**

Isabel M.D. Rosa^1,4^, Cristina Gabriel^2^, Joāo M.B. Carreiras^3^

^1^ Life Sciences Department, Imperial College of London, Silwood Park Campus, Buckhurst Road, Ascot SL5 7PY, United Kingdom.

^2^ Centro de Ecologia Aplicada Prof. Baeta Neves, Instituto Superior de Agronomia, Tapada da Ajuda, 1349 - 017 Lisboa, Portugal, [cristinagarciagabriel@gmail.com](mailto:cristinagarciagabriel@gmail.com)

^3^ National Centre for Earth Observation (NCEO), University of Sheffield, Hicks Building, Hounsfield Road, Sheffield S3 7RH, United Kingdom, [j.carreiras@sheffield.ac.uk](mailto:j.carreiras@sheffield.ac.uk)

^4^ Biodiversity Conservation Group, German Centre for Integrative Biodiversity Research (iDiv), Deutscher Pl. 5E, 04103 Leipzig, Germany, [isabel.rosa@idiv.de](mailto:isabel.rosa@idiv.de)

**Corresponding author:** Dr. Isabel M.D. Rosa

E-mail: isabel.rosa@idiv.de

Telephone number: +49 (0) 341 9733229

Postal address**:** Biodiversity Conservation Group, German Centre for Integrative Biodiversity Research (iDiv), Deutscher Pl. 5E, 04103 Leipzig, Germany.

**Running title:** Fragmentation in the Amazon

**Supplementary Tables**

**Table S1 –** Summarised information regarding the three landscapes studied.

|  | **Machadinho d’Oeste**  **(settled in 1980s)** | | **Santarém**  **(settled in 1970s)** | | | **Manaus**  **(settled in 1970s)** | |
| --- | --- | --- | --- | --- | --- | --- | --- |
|  | **1984** | **2011** | **1984** | **2010** | | **1985** | **2011** |
| Mature Forest Cover (%) | 90.7 | 31.6 | 78.8 | 46.2 | | 83.2 | 71.9 |
| Non-Forest Cover (%) | 0.6 | 32.6 | 2.0 | 9.7 | | 8.4 | 8.9 |
| Secondary Forest Cover (%) | 8.7 | 35.8 | 19.2 | 44.1 | | 8.3 | 19.3 |
| Permanent Crops (%)* | 0.30 | 1.20 | 0 | 0.03 | | 0.07 | 0.13 |
| Annual Crops (%)* | 0.93 | 1.19 | 0 | 1.16 | | 0.06 | 0.05 |
| Cattle density (heads/ha) | 0 | 0.29 | 0 | 0.01 | | 0.01 | 0.01 |
| % of landscape occupied by pastures in 2014 | 40.2 | | 12.1 | | | 1.2 | |
| Mature Forest (%)  inside CUs | 23.4 | 55.7 | 40.0 | | 62.2 | 45.0 | 46.7 |
| Non-Forest (%)  inside CUs | 0.8 | 2.0 | 13.9 | | 7.3 | 30.5 | 35.9 |
| Secondary Forest (%)  inside CUs | 23.1 | 13.9 | 26.4 | | 16.7 | 34.9 | 32.5 |
| % of conservation units’ area occupied by Mature Forest | 91.3 | 75.7 | 85.4 | | 78.0 | 87.3 | 78.0 |
| % of conservation units’ area occupied by Non-Forest | 0.0 | 2.9 | 0.8 | | 2.0 | 6.0 | 7.4 |
| % of conservation units’ area occupied by  Secondary Forest | 8.7 | 21.4 | 13.8 | | 20.0 | 6.7 | 14.6 |

*data for permanent and annual crop harvesting starts in 1990.

**Table S2** – Landsat Thematic Mapper (TM) and Enhanced TM plus (ETM+) data available for the three sites (adapted from Carreiras et al. (2014)).

| **Machadinho d'Oeste** | | **Santarém** | | **Manaus** |  |
| --- | --- | --- | --- | --- | --- |
| **Date^1^** | **Sensor** | **Date^1^** | **Sensor** | **Date^1^** | **Sensor** |
| 19840617 | TM | 19840824 | TM | 19850604 | TM |
| 19860810 | TM | 19850726 | TM | 19880815 | TM |
| 19870712 | TM | 19860729 | TM | 19890802 | TM |
| 19890717 | TM | 19870716 | TM | 19910808 | TM |
| 19900618 | TM | 19880803 | TM | 19920607 | TM |
| 19910925 | TM | 19890822 | TM | 19941019 | TM |
| 19940816 | TM | 19900809 | TM | 19950920 | TM |
| 19950803 | TM | 19910711 | TM | 19960720 | TM |
| 19960704 | TM | 19931020 | TM | 19990713 | TM |
| 19970723 | TM | 19951010 | TM | 20010827 | ETM+ |
| 19980624 | TM | 19960825 | TM | 20020830 | ETM+ |
| 19990729 | TM | 19970727 | TM | 20030809 | TM |
| 20010803 | TM | 19980815 | TM | 20060716 | TM |
| 20030724 | TM | 19990903 | TM | 20070804 | TM |
| 20050713 | TM | 20000905 | TM | 20080806 | TM |
| 20060716 | TM | 20010916 | ETM+ | 20090910 | TM |
| 20070703 | TM | 20030829 | TM | 20100727 | TM |
| 20080806 | TM | 20050701 | TM | 20110831 | TM |
| 20090809 | TM | 20060805 | TM |  |  |
| 20100625 | TM | 20070621 | TM |  |  |
| 20110612 | TM | 20081130 | TM |  |  |
|  |  | 20090712 | TM |  |  |
|  |  | 20100629 | TM |  |  |

^1^Date format is yyyymmdd**Table S3 –** Parameters of the linear models adjusted for each region (Machadinho d’Oeste (MO), Santarém (ST) and Manaus (MA)), for each metric (edge density (ED), clumpiness index (CLUMPY), area-weighted mean patch size (AREA_AM) and area-weighted mean patch shape index (SHAPE_AM)), and for each land cover class (mature forest (MF), non-forest (NF) and secondary forest (SF)), inside or outside conservation units (CUs), taking into account temporal autocorrelation.

|  |  |  | **Inside CUs** | | | **Outside CUs** | | |
| --- | --- | --- | --- | --- | --- | --- | --- | --- |
| **Metric** | **Study**  **Area** | **Land Cover** | **Slope** | **Std. Error** | **p-value** | **Slope** | **Std. Error** | **p-value** |
| ED | MO | MF | 0.08 | 0.01 | <0.001 | 0.34 | 0.11 | 0.01 |
|  |  | NF | 0.07 | 0.01 | <0.001 | 0.83 | 0.07 | <0.001 |
|  |  | SF | 0.16 | 0.04 | 0.001 | 1.49 | 0.17 | <0.001 |
|  | ST | MF | 0.39 | 0.04 | <0.001 | 0.92 | 0.16 | <0.001 |
|  |  | NF | 0.02 | 0.12 | 0.89 | 0.11 | 0.46 | 0.808 |
|  |  | SF | 0.50 | 0.09 | <0.001 | 1.19 | 0.30 | <0.001 |
|  | MA | MF | 0.21 | 0.01 | <0.001 | 0.08 | 0.01 | <0.001 |
|  |  | NF | 0.06 | 0.02 | 0.001 | -0.10 | 0.04 | 0.02 |
|  |  | SF | 0.24 | 0.01 | <0.001 | 0.39 | 0.19 | 0.05 |
| CLUMPY | MO | MF | -2 x10^-4^ | 3 x10^-5^ | <0.001 | -0.003 | 1 x10^-4^ | <0.001 |
|  |  | NF | 0.01 | 0.002 | <0.001 | 0.004 | 0.001 | 0.001 |
|  |  | SF | 0.01 | 0.005 | 0.03 | 0.01 | 0.003 | 0.04 |
|  | ST | MF | -0.001 | 1 x10^-4^ | <0.001 | -0.01 | 0.001 | <0.001 |
|  |  | NF | 0.01 | 0.002 | 0.03 | 0.01 | 0.002 | 0.01 |
|  |  | SF | 5 x10^-4^ | 0.001 | 0.55 | 3 x10^-4^ | 0.001 | 0.79 |
|  | MA | MF | -0.001 | 2 x10^-5^ | <0.001 | -2 x10^-4^ | 3 x10^-5^ | <0.001 |
|  |  | NF | 7 x10^-5^ | 0.003 | 0.98 | 0.001 | 0.001 | 0.18 |
|  |  | SF | 0.002 | 0.001 | 0.01 | 0.01 | 0.001 | <0.001 |

**Table S3 – (cont.)**

| **Metric** | **Study**  **Area** | **Land Cover** | **Slope** | **Std. Error** | **p-value** | **Slope** | **Std. Error** | **p-value** |
| --- | --- | --- | --- | --- | --- | --- | --- | --- |
| Area_AM | MO | MF | -107 | 19.4 | <0.001 | -2478 | 367.5 | <0.001 |
|  |  | NF | 9.1 | 1.3 | <0.001 | 187.8 | 69.0 | 0.01 |
|  |  | SF | 7.0 | 2.3 | 0.01 | 23.9 | 8.1 | 0.01 |
|  | ST | MF | -1085 | 225.5 | <0.001 | -1865 | 198.0 | <0.001 |
|  |  | NF | 33.7 | 30.4 | 0.28 | 35.0 | 36.0 | 0.34 |
|  |  | SF | 293 | 72.4 | <0.001 | 885.0 | 122.6 | <0.001 |
|  | MA | MF | -2175 | 358.9 | <0.001 | -4708 | 1266 | 0.002 |
|  |  | NF | 2.02 | 3.7 | 0.59 | -2.74 | 11.0 | 0.81 |
|  |  | SF | 12.0 | 2.7 | <0.001 | 159.84 | 89.6 | 0.09 |
| Shape_AM | MO | MF | 0.03 | 0.01 | <0.001 | -0.37 | 0.06 | <0.001 |
|  |  | NF | 0.04 | 0.01 | <0.001 | 0.24 | 0.05 | <0.001 |
|  |  | SF | 0.10 | 0.02 | <0.001 | 0.23 | 0.05 | <0.001 |
|  | ST | MF | 0.19 | 0.03 | <0.001 | 0.10 | 0.04 | 0.01 |
|  |  | NF | 0.07 | 0.12 | 0.59 | 0.06 | 0.29 | 0.82 |
|  |  | SF | 0.73 | 0.23 | 0.004 | 1.67 | 0.26 | <0.001 |
|  | MA | MF | 0.12 | 0.05 | 0.05 | -0.35 | 0.10 | 0.002 |
|  |  | NF | 0.06 | 0.03 | 0.11 | -0.07 | 0.06 | 0.30 |
|  |  | SF | 0.04 | 0.02 | 0.04 | 0.26 | 0.24 | 0.28 |

**Table S4 –** Parameters of the linear models adjusted for each region (Machadinho d’Oeste, Santarem and Manaus), taking into account temporally autocorrelated errors, to investigate positive/negative trends over time of the relative incidence of deforestation (RID) on each type (County, State, or Federal) of conservation unit (CU), or outside these units (Non-CU).

| **Study**  **Area** | **Conservation**  **Status** | **Slope** | **Std.**  **Error** | **p-**  **value** |
| --- | --- | --- | --- | --- |
| Machadinho d’Oeste | Non – CU | -0.002 | 3 x10^-4^ | < 0.001 |
|  | State – CU | 0.01 | 0.001 | < 0.001 |
| Santarém | Non – CU | 0.003 | 0.001 | < 0.001 |
|  | Federal – CU | -0.004 | 0.001 | < 0.001 |
| Manaus | Non – CU | -0.01 | 4 x10^-4^ | < 0.001 |
|  | County – CU | -0.003 | 0.001 | < 0.001 |
|  | State – CU | 0.01 | 0.001 | < 0.001 |
|  | Federal – CU | -0.06 | 0.01 | < 0.001 |

**Supplementary Figures**

**
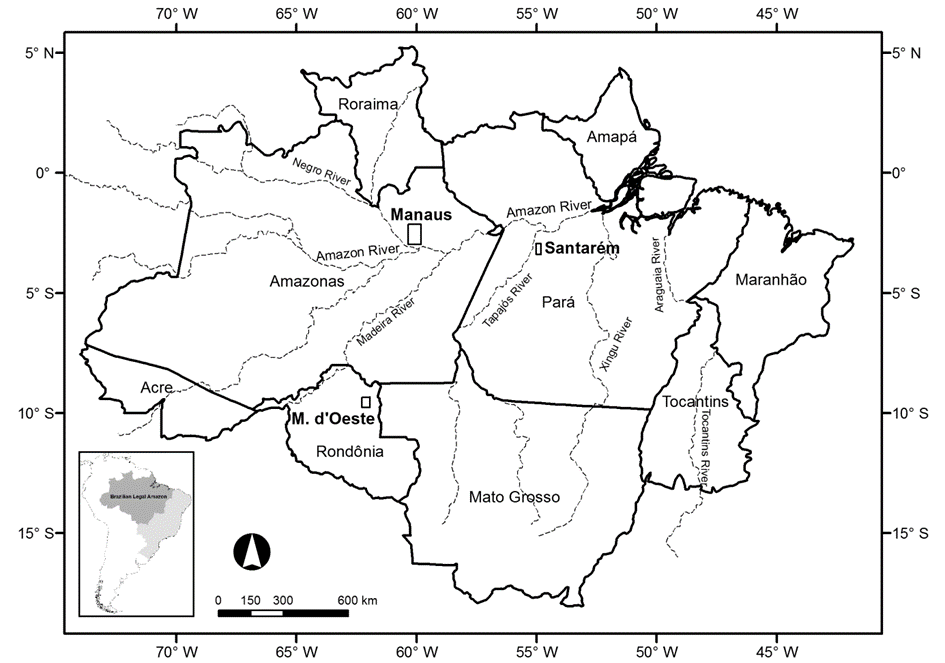
**

**Figure S1** – Location of the three study sites: Manaus, Santarém and Machadinho d’Oeste (M. d’Oeste) in the Brazilian Legal Amazon; also shown are the states boundaries and major rivers in the Amazon basin (adapted from Carreiras et al. (2014)).

**

**

**Figure S2** – Relative incidence of deforestation (%) on areas without conservation units (Non CU) and per type of conservation unit – federal, state or county – in Machadinho d’Oeste (top), Santarém (middle) and Manaus (bottom) from 1984 through 2011. The cross(es) indicates the year of creation of the conservation units, when no cross is present it means the units already existed at the beginning of the time series covered in this study (e.g. Tapajós National Forest in Santarém was designated in 1974).


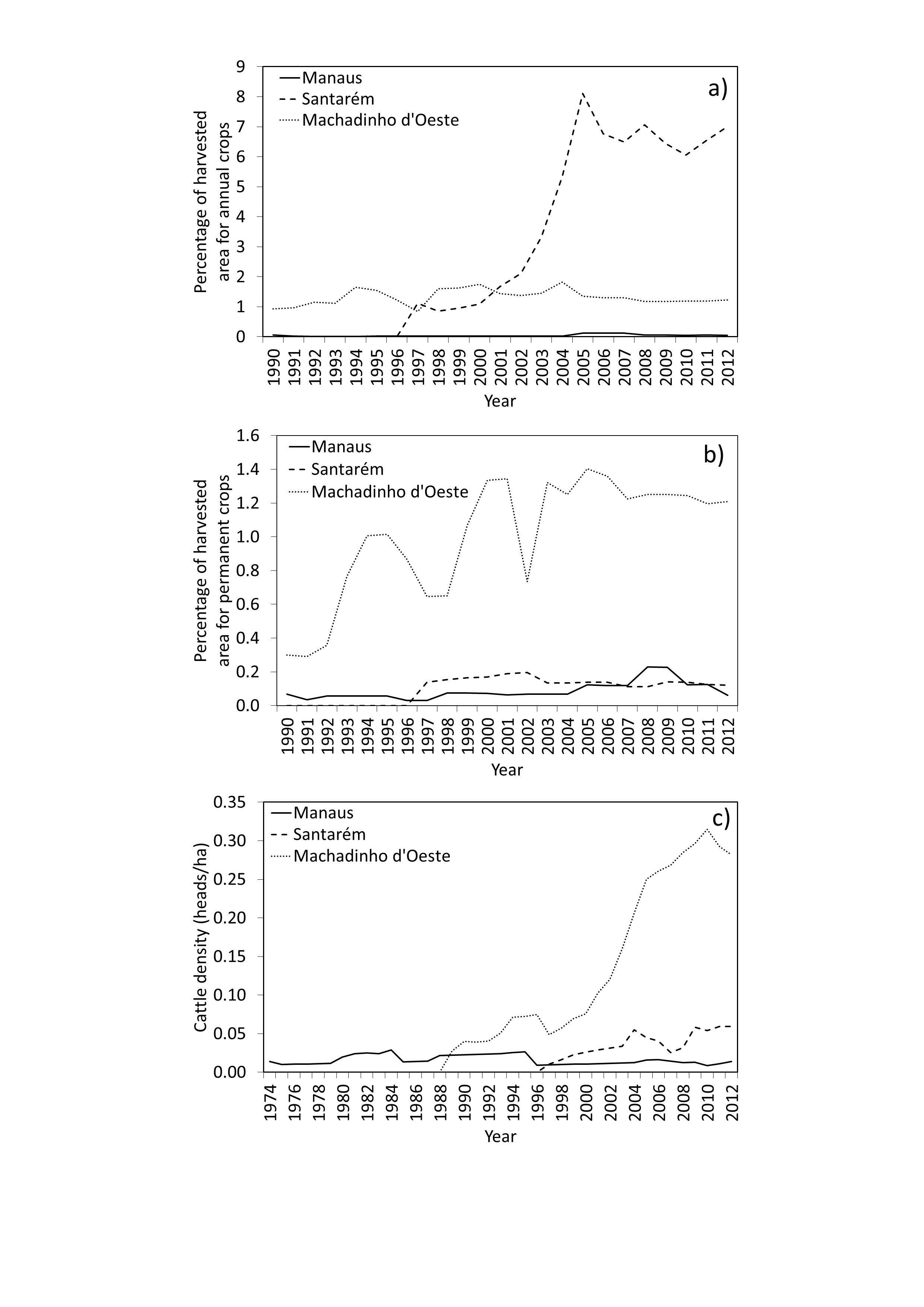


**Figure S3** – Time series of agricultural data from IBGE: a) percentage area harvested of annual crops between 1990 and 2012 (area harvested for annual crops divided by the municipality area), b) percentage area harvested of permanent crops between 1990 and 2012 (area harvested for permanent crops divided by the municipality area), and c) density of cattle heads in the period 1974-2012 (cattle heads divided by the municipality area) in Manaus, Santarém and Machadinho d’Oeste.
